# “Telling me not to worry…” Hyperscanning and Neural Dynamics of Emotion Processing During Guided Imagery and Music

**DOI:** 10.3389/fpsyg.2019.01561

**Published:** 2019-07-25

**Authors:** Jörg C. Fachner, Clemens Maidhof, Denise Grocke, Inge Nygaard Pedersen, Gro Trondalen, Gerhard Tucek, Lars O. Bonde

**Affiliations:** ^1^Cambridge Institute for Music Therapy Research, Anglia Ruskin University, Cambridge, United Kingdom; ^2^Josef Ressel Centre for Personalised Music Therapy, IMC University of Applied Sciences Krems, Krems an der Donau, Austria; ^3^Melbourne Conservatorium of Music, University of Melbourne, Melbourne, VIC, Australia; ^4^Department of Communication and Psychology, The Faculty of Humanities, Aalborg University, Aalborg, Denmark; ^5^Centre for Research in Music and Health, Norwegian Academy of Music, Oslo, Norway

**Keywords:** music therapy, moments of interest, dyadic interaction, imagery, emotion, EEG, alpha asymmetry, social neuroscience

## Abstract

To analyze how emotions and imagery are shared, processed and recognized in Guided Imagery and Music, we measured the brain activity of an experienced therapist (“Guide”) and client (“Traveler”) with dual-EEG in a real therapy session about potential death of family members. Synchronously with the EEG, the session was video-taped and then micro-analyzed. Four raters identified therapeutically important moments of interest (MOI) and no-interest (MONI) which were transcribed and annotated. Several indices of emotion- and imagery-related processing were analyzed: frontal and parietal alpha asymmetry, frontal midline theta, and occipital alpha activity. Session ratings showed overlaps across all raters, confirming the importance of these MOIs, which showed different cortical activity in visual areas compared to resting-state. MOI 1 was a pivotal moment including an important imagery with a message of hope from a close family member, while in the second MOI the Traveler sent a message to an unborn baby. Generally, results seemed to indicate that the emotions of Traveler and Guide during important moments were not positive, pleasurably or relaxed when compared to resting-state, confirming both were dealing with negative emotions and anxiety that had to be contained in the interpersonal process. However, the temporal dynamics of emotion-related markers suggested shifts in emotional valence and intensity during these important, personally meaningful moments; for example, during receiving the message of hope, an increase of frontal alpha asymmetry was observed, reflecting increased positive emotional processing. EEG source localization during the message suggested a peak activation in left middle temporal gyrus. Interestingly, peaks in emotional markers in the Guide partly paralleled the Traveler's peaks; for example, during the Guide's strong feeling of mutuality in MOI 2, the time series of frontal alpha asymmetries showed a significant cross-correlation, indicating similar emotional processing in Traveler and Guide. Investigating the moment-to-moment interaction in music therapy showed how asymmetry peaks align with the situated cognition of Traveler and Guide along the emotional contour of the music, representing the highs and lows during the therapy process. Combining dual-EEG with detailed audiovisual and qualitative data seems to be a promising approach for further research into music therapy.

## Introduction

Musical imagery research investigates imagination of intervals, melodies and other musical elements in order to compare them to the listening process (Hubbard, 2010). This may include any imagery of sound and music where there is no physical source, e.g. when conductors study scores or composers compose without a piano, or when participants are asked to recall and imagine what they've heard before (Schaefer et al., 2011). However, in this explorative study, we were primarily interested in shared visual imagery and emotion related to music listening occurring in a therapeutic setting with a therapist and a client, while the client is in an altered state of consciousness (ASC). Listening to music can completely absorb people, cutting off other sensory input, but absorption skills seem to be linked to music preference, imagery, hypnotizable and intensity of emotions evoked (Snodgrass and Lynn, 1989; Kreutz et al., 2008; Schäfer et al., 2013). The goal of this case study was to test a series of subsequent propositions regarding emotion-, imagery-, and event-related activity in frontal and parietal regions of the brain.

### Spontaneous Imagery, Emotion and Gaining Insight

In the Bonny Method of Guided Imagery and Music (BMGIM or here GIM), a client (in GIM often referred to as “the Traveler” doing an imaginary “journey,” while the therapist is referred to as “the Guide”) describes images, feelings, or thoughts that occur spontaneously while eyes-closed listening to special music programs in an induced ASC (Bonny, 2002). Imagery or image refers to:

“*experiences of music during the listening phase of BMGIM, including images in all sensory modalities, kinesthetic images, body sensations, feelings, thoughts and noetic images (an intuitive sense of imaginal events that arise outside of other imagery modes).”* (Goldberg, 2002, p. 360).

Commonly, certain passages during the imagery process will have pivotal meaning for the Traveler and become a focus in the therapy process (Grocke, 1999). A pivotal moment is a turning point in which a client makes a transition. Getting in touch with one's own emotions and gaining personal insight during such moments in the imagery process seems to be a driving force of change (Maack and Nolan, 1999). Specifically, “gaining new insight” or discovery of new perspectives is considered to be an important theme during such “transforming” moments (Lin et al., 2010), although the imagery itself is diverse and multimodal, including visual, auditory, somatic, direct memories, involuntary and unbidden imagery, images of significant people, places and events from the person's history (Bonde, 2019). For an overview of imagery categories in GIM see (Grocke, 1999, p. 15).

### Spontaneous Imagery and Interactive Processes

A typical GIM session comprises an initial discussion of the Traveler's concerns, and defining a focus for the music and imagery experience. The Guide provides a relaxation induction for the Traveler who reclines with eyes closed, chooses a pre-determined music program, or spontaneously chooses music to match the Traveler's imagery. As the music plays the Traveler describes any imagery, feeling, or thoughts, while the Guide facilitates occasionally with non-direct inquiries. Bruscia defined the Bonny method of GIM as

*“an individual form of exploring consciousness, which involves *spontaneous imaging* in an expanded state of consciousness, to pre-designed programs of classical music, *while interacting with a guide* who uses non-directive, non-analytical, music-based interventions within a client-centered orientation, …” (Bruscia, 2002, p. 46; italics from author)*.

Of interest for this research is engaging in the client-therapist dialogue during the spontaneously occurring imagery during music listening in an ASC. The client is not just reporting on what he or she “sees” during the imagery process, it is surrendering to an intense process (Blom, 2011, 2014), it is a “lived experience during the imagery … that occurs as the client interacts with the images” (Heinschel, 2002, p. 332) facilitated by the therapist as a “personalized response” (ibid. p. 326) during the therapy process.

In this research, we wanted to study the interaction between a client and a therapist during a real-world GIM session, in which a therapist accompanies and interacts to deepen the experience of the client's emerging images. We were interested in how spontaneously evoked and guided imagery is processed and shared with the therapist and investigated the temporal dynamics of emotional processing with EEG during imagery of important moments in GIM. Spontaneous imagery is essential for the therapeutic process (Trondalen, 2016), but how meaningful imagery emerges in the time-course of the therapy and how its temporal dynamics relate to emotional processing have not been investigated so far. Many randomized controlled trials showed that music therapy works, but how it works remains unclear (Maratos et al., 2011). As the therapeutic relationship is essential to music therapy action, investigating brain correlates of dyadic therapeutic processes is crucial to understand and explain how music therapy works (Fachner, 2014; Hunt, 2015). As dyadic recordings have not been done before in a music therapy context (and especially in GIM), this the first EEG hyperscanning study investigating shared emotional processing and temporal dynamics of its neural correlates.

#### GIM and Temporal Neural Dynamics of Affective States

Alan Lem, in a first but technically limited attempt to capture the continuous GIM process with EEG, music analysis and verbal reports, described frontal responses (slowing of the EEG and increase of amplitude) during distinct music passages, relaxation and occurrence of visual imagery indicating a “specific time of occurrence of a particular imagery response” (Lem, 1998, p. 15). A formal music analysis and profiling the music's affective expression divided the piece (*G. Pierne's Concertstück for harp and orchestra*) into 26 segments within 3 larger sections. EEG amplitude and frequency were synchronously plotted against the wave form of the music and correlations were explored. After an initial EEG slowing in the first section, fluctuations in the latter 2 sections, and an association between EEG bursts and reported occurrence of visual imagery at the end of the music's section 1 and 2 (see p. 9), several segment specific observations were described.

Of interest for us here is that the reported “correlations and associations” seem to convey a temporal dynamic between the identified affective expressions of the piece, the state changes as indicated in the frontal EEG and the corresponding imagery processes along the timeline of the GIM process of the 27 participants. The EEG changes are pointing to what Helen Bonny coined as the guiding “affective contour” (Bonny, 1978, p. 39), which is determined through the music and gives the therapist a more or less predictive tool to elicit particular emotions within an interactive narrative structure of the session.

Hunt (2011) went a step further in analyzing the imagery process, in a quasi-experimental condition, during a controlled Music & Imagery session and analyzed responses to 6 probe modalities of the imagery experience (visual, kinesthetic, body, affect, memory and also interaction). Hunt provided a verbal probe for each of the categories and then analyzed the following 10 s of EEG. Additionally, she interviewed the participants about their experience based on a video of their session. In the interactive imagery condition (a probe of dialoguing with an imaginary person), stronger coherence was observed relating to localizations of the language centers (Broca and Wernicke) and a general observation was that “beta and gamma frequencies played a significant role in how participants … made meaning out of the imagery” (Hunt, 2011, p. III). This may give a hint on how clients integrate new information and meaning of the imagery.

A neurometric GIM single case study of a GIM Traveler by Fachner et al. (2015) described distinct neurometric resting-state and ASC induction differences. ASC exhibited more Alpha z-score deviations than the resting-state when comparing the single case against a normative database. In addition, exploring source localization patterns of an important imagery segment of the therapy session returned highest cortical activation in cuneus and pre-cuneus. However, how the emotional involvement during the imagery can be studied and linked to the interaction of therapist and client is still an open question.

#### Alpha Band, Emotion and Imagery

Schaefer et al. (2011) investigated alpha band signatures induced by imagining and perceiving short natural, well-known musical phrases. Here imagery was interpreted as internally directed attention that would inhibit activity in visual areas bilaterally distributed in parieto-occipital areas of the brain. Indeed, participants exhibited individually shifting but increased parieto-occipital alpha power topographies while imagining music. Moreover, Cooper et al. (2003) described frontal and occipital alpha responses while imagining pure tones and discussed increased alpha power not as idling but as an “index [of] active inhibition of non-task relevant cortical areas” (p. 73). In other words, if subjects turn their attention inwardly and start imagining, then alpha power indexes those parts in the brain topography that are actively inhibited (or idling) and thus show less cortical activity.

However, with regards to emotional responses evoked by music, particularly alpha power over frontal brain areas seems to indicate positive or negative emotions. Schmidt and Trainor (2001) showed that frontal alpha power distinguished the valence of musical excerpts. Participants exhibited greater relative left frontal EEG activity to “joy” and “happy” musical excerpts and greater relative right frontal EEG activity to fear and sad musical excerpts. When listening to music rated as representing positive valence, significant left frontal activity changes in DC-EEG were found (Altenmuller et al., 2002). Mikutta et al. (2012, p. 423) “aimed to identify the physiological correlates of continuous changes in subjective emotional states” while listening to a longer piece of music. They interpreted the observed right-frontal suppression of alpha activity as being related to the emotional content independent of the dynamics expressed in loudness parameters. However, although subjective ratings were continuously recorded, temporal dynamics of the correlations and accordingly changes in the frontal processing were not investigated. Interestingly, Jäncke et al. (2015) presented an aria repeatedly to 16 participants and described “a remarkable variability in EEG oscillations, both within and between the repeated presentations of the aria” (p. 1) and an increase of event-related synchronization in all frequency bands. They concluded that the state of listening and attention on internal processes may best be characterized as mind wandering but “that the neurophysiological activations occurring during music listening are dynamic and not stationary” (ibid.).

## Aims

In this research we aim to explore spontaneous emerging imagery and how this is emotionally processed in a dyadic therapeutic relationship. We are interested how imagery and emotion processing are interacting in the therapeutic process and how the corresponding time series of neural correlates is related to the identified events in particular moments of therapeutic interest. As healing processes are known to deepen and intensify during particular moments in which meaningful events are situating cognitive processes, we aim to analyze authentic data of a music therapy process (Fachner, 2016, 2017).

In therapy, therapists and clients are immersed through the senses, interaction, embodiment and narrative experience in real-time contextually performed interpersonal action. Clinical experience and qualitative research suggests that *spontaneously emerging imagery* in the client is recognized by the therapist, but also from both as a mutual spontaneously occurring feeling (Blom, 2011, 2014). Such events may be accompanied with an unspecific emotion, recognizing a spontaneous occurring thought, making the therapist sense that “something” in the client has happened (Blom, 2014; Trondalen, 2016, 2019). Indices of such events may include a range of non-verbal indices and - as Rothschild (1962) and Watzlawick et al. (2014) would frame it - biosemiotic interpunctions of the internal process, for example a change of prosodic features while talking, a different toning and intentionality of body posture, a change of facial expression and gesture and other context-related indicators of emotional processing. These indices may occur as serial and parallel biosemiotic epiphenomena of the internal imagery processes. Rothschild was interested in a system of semiotic symbols transmitted and received via bodily processes, while Watzlawick discussed that during interaction we can see a time series of interpunctions, i.e., sequences or moments in the communicative process that define the quality of the relationship. Therapists are trained to recognize such processes while they also become aware of sudden ideas which are not usually their own, and would interpret this as material that they recognize from the client:

“*The therapist was sensitive to small and subtle changes in the patient's gestures, pace, intensity and tone of voice, body language and facial expression. During the sessions, any changes in patients' language expressing imagery and their personal challenges or problems were observed. Gaining new insight, the patients attempted to change or control their negative emotions…” (Lin et al.*, *2010**, p. 1146)*.

Recording a dual-EEG of a Traveler and a Guide in a GIM session may capture such emergence of spontaneous imagery with meaningful emotional content and allow for analysis of its time course.

### Spontaneous Imagery, Music, Shared Emotional States and Therapeutic Change

“Unexpected change in musical features intensity and tempo—and thereby enhanced tension and anticipation—is proposed to be one of the primary mechanisms by which music induces a strong emotional response in listeners” (Arjmand et al., 2017, p. 1). Other research on musical emotions supports this proposition (Huron, 2006; Juslin, 2013; Wärja and Bonde, 2014; Koelsch, 2015). However, Arjmand and colleagues investigated subjective and averaged, as well as temporal dynamics of EEG frontal asymmetry (FA) measures of experienced emotion of participants listening to self-selected music. They described a left pre-frontal response (indicating positive emotions) during pleasurable music but also peak FA responses “to co-occur with key musical events relating to change” (ibid).

However, a GIM session is not solely focusing on the music, but there is a “healing contract” (Fachner, 2017); that is, a therapeutic relationship is established between the Traveler and the Guide, who uses both the occurring imagery for psychotherapeutic purposes while verbally interacting to deepen the experience of the emerging images. According to the *shared network hypothesis* “observing or imaging another in a particular affective state activates a representation of the same state in the observer” (Acquadro et al., 2016, p. 7), while also activating similar emotion-specific networks with a “specificity in the temporal flow of the affect information from the sender to the perceiver” (ibid). In other words, there are moments in which we recognize and share meaningful emotional states in and with others and this seems to be mediated by shared neural representations and brain-to-brain coupling processes; the latter seem to be signified by increased theta- and alpha power cross-correlations (for an overview, see Acquadro et al., 2016).

However, to find out about the meaning, the timing (synchronously or interpunctive) and the (sensory) mode of the emerging imagery (visual, kinesthetic, tactile, somatic gustatory, olfactory, auditory but in a therapeutic context also affect, memory, interaction; see Hunt, 2011), we need to know about its specific content and importance for the therapeutic process at a particular time-point during the therapy session.

#### Moment of Interest (MOI) Identification

Usually therapists select particular parts from one or a series of sessions to describe a narrative of therapeutic change processes in therapy (Bruscia, 1991; Hibben, 1999; Aldridge, 2004; Bonde, 2004). In a case study, there is usually one first-person perspective (from the therapist) on the therapy process and a selection of important and thus interesting episodes of a session or a series of sessions (Grocke and Moe, 2015; Hunt, 2016). These *moments of interest* may be indicative of a particular string of therapy progress, describe encounter between therapist and client, or represent pivotal change of behavior following the emergence of a particular important imagery and insight. In other words, therapists rate some segments of therapy to be of indicative importance for clients' development in therapy. Moments of interest (MOI) refer to the selected target sequences in a time series of clinically relevant events that are recognized and selected based on personal and/or professional preferences and interests (Fachner, 2017).

To analyze the particular parts of the process in more detail, microanalytic approaches to music therapy (Wosch and Wigran, 2007) utilize the selections and ratings of other therapists or non-therapists and try to achieve a degree of content validity of the chosen parts. Because asking only the Guide might lead to missing other aspects of the therapy (Spiro and Himberg, 2016), we asked both the Guide and the Traveler for their MOIs and additionally requested MOI selections from two other GIM therapy experts. If there would be important moments indicating change in the therapy progress, then these moments should be selected by all raters, resulting in an overlap of their selections, thus indicating a higher degree of content validity.

Utilizing an objectivist case study approach (Ridder and Fachner, 2016), we studied these MOIs and how they are contextualized in the overall progression of the therapy with a set of objective data, namely an EEG, which has an objective reality in itself, and allows inferences and propositions about the time series data of brain processing of both therapist and client.

#### Brain Activity and Asymmetries

Davidson (2004) attributed a leading role in processing emotion to the function of the frontal cortex regulating approach/withdrawal behavior. Thus, positive emotion and approach behavior would be processed in the left, while negative emotions and withdrawal would be linked to right frontal lobe activity (Harmon-Jones, 2003). According to Davidson, EEG activity reflects this activity on the cortical level as measured in alpha asymmetries at frontal leads, and for depression most prominently on the pre-frontal leads F3/4 (Gold et al., 2013), i.e., frontal alpha asymmetry (FAA). FAA is a well-established measure of emotional processing (Harmon-Jones et al., 2010; Smith et al., 2017) and offers a simple connectivity ratio (ln F4- ln F3) between the homolog electrode parameters of the left and right frontal lobe. Positive values indicate dominant activity on the left frontal cortical side, while negative values vice versa. FAA and frontal measures indicated positive or negative valence of musical emotions (Field et al., 1998; Schmidt and Trainor, 2001; Altenmuller et al., 2002).

Heller and Nitscke (1997) introduced asymmetries of parietal alpha power indicating arousal and intensity of emotional processing, i.e., increased right parietal activity would indicate the arousal and therefore the intensity of emotions experienced (Schmidt and Trainor, 2001; Jakobi, 2009). Valence and arousal are discussed to represent the intensity of perceived and felt musical emotions (Eerola and Vuoskoski, 2013). However, parietal alpha asymmetries (PAA) are also discussed to indicate anxiety and withdrawal, but with inconclusive results (Stewart et al., 2011).

FAA and frontal biomarkers have been successfully applied in music therapy research correlating therapy effectiveness and brain activity changes in depression (Fachner et al., 2013), cancer and palliative care (Lee et al., 2012; Ramirez et al., 2018) and Disorders of Consciousness (O'Kelly et al., 2013). Comparing the effect of music therapy on depressed patients' resting-state EEG, frontal brain activity, as indicated in alpha power and asymmetries, was significantly different to those not receiving music therapy. Further, Frontal Midline Theta (FMT) correlated positively with the reduction of anxiety measures after music therapy (Fachner et al., 2013).

FMT has been utilized as an anxiety marker for anxiolytics (Mizuki et al., 1989; Mitchell et al., 2008). Increased FMT power is related to lower levels of anxiety, and has indicated states of internalized attention and positive emotional experience (Aftanas and Golocheikine, 2001). In music research comparing non- / preferred and non- / pleasurable music, an increase of FMT along the listening of pleasurable preferred music was observed (Sammler et al., 2007). Recently another study confirmed FMT increases over the time-course of listening to pleasurable music (Nemati et al., 2019).

Continuous measures of frontal activities seem also to be indicative of leading and following in social interaction (Konvalinka et al., 2014) and during duet guitar improvisation (Müller et al., 2013). Furthermore, mobile EEG applications allow authentic *in situ* recordings (Debener et al., 2012) and complex data integration (Maidhof et al., 2014). In short, frontal EEG measures are sufficient and promising tools to research therapy processes in real-world settings of music therapy (Fachner, 2016).

In this study, we were interested how the temporal FAA dynamics change according to the emotional impact of the emerging imagery and how this is related to the therapy process, whereby analysis of temporal FAA dynamics focuses on the asymmetry peaks in the time series (Allen and Cohen, 2010). In our study, we expect peaks to appear during important MOI segments and we will study the biosemiotic interpunctions of the peaks in those segments.

### Propositions Regarding Emotional Valence and Arousal During Selected MOIs

#### Emotion-Related Activity

Based on the studies discussed in above sections on “Spontaneous Imagery and Interactive Processes” and “Brain Activity and Asymmetries,” we will explore the differences in several markers of emotional processing.

I. We expect differences between emotional processing during important moments and during the resting-state, and thus a difference in the alpha asymmetry (AA) measures.
  - More specifically, compared to the resting-state, we expect during MOIs more left-shifted frontal AA (see Field et al., 1998), possibly indicating positive emotional valence, and more right-shifted parietal AA, possibly indicating emotional intensity/arousal (see Schmidt and Trainor, 2001).
II. We expect FMT power to increase during positive emotional processing (Aftanas and Golocheikine, 2001; Sammler et al., 2007).

#### Imagery-Related Activity

As discussed in section “Alpha Band, Emotion and Imagery,” increased alpha power may index those brain areas that are less active during imagery and when attention is turned inwardly (see Cooper et al., 2003; Schaefer et al., 2011).

III. We expect the visual cortex to be more active (to exhibit less alpha power) during the imagery process than during the resting-state.
IV. Additionally, during GIM (compared to rest), we expect that temporo-parietal areas are less active (i.e. more alpha power) then occipital areas.

#### Event-Related Activity

In the analysis of the temporal dynamics of emotional processing we will inspect the moment-to-moment interaction and the co-occurring event-related F/PAA during interesting moments.

V. We expect strong peaks to occur within imagery events of strong emotional valence (see Arjmand et al., 2017).
VI. We expect the peaks to indicate a temporal dynamic of dyadic biosemiotic interpunction of the therapy process, i.e., peaks indicating shared emotional processing (see Acquadro et al., 2016).

## Materials and Methods

### Participants

Two right-handed, female healthy participants (age: 72 and 65 years old) took part in the EEG study. One of them was the Guide (therapist) and the other the Traveler (client), both with more than 20 years of experience in music therapy. Two experienced GIM therapists (age: 62 and 67 years) with about 20 years of GIM practice watched the video-recorded session and selected important moments of interest.

### Therapy Background

The Traveler reported to the Guide that her son's pregnant girlfriend had recently been diagnosed with a life-threatening illness and that therefore the birth of the (grand) child was in danger. An operation had been put on hold to keep the child healthy. The Traveler's motivation for the therapy was to find out (1) how to cope with the helplessness and anxiety of losing the child and (2) how to keep hope. The intentions of the Guide were to (1) be present to the Traveler's situation and (2) provide a supportive music experience that would contain the Traveler while exploring strong emotions. Both Traveler and Guide are experienced therapists with more than 30 years of clinical practice in music therapy and the Bonny Method of GIM and are colleagues. Nevertheless, a therapy healing contract was established, as the Traveler was asking for help with an actual personal problem.

Participants gave informed written consent prior to the study (including written informed consent for the publication of their identifiable data), which was approved by the local ethics committee of Anglia Ruskin University and conducted in accordance with the Declaration of Helsinki.

### Materials and Apparatus

#### Procedure

Before and after the therapy session, a resting-state EEG was recorded for ca. 4 min. while participants had their eyes closed (for EEG details, see below). During the therapy, the Guide had her eyes open while the Traveler had her eyes closed.

The session followed the usual structure. It began with a pre-talk (ca. 12 min) in which the Traveler described a situation she wanted to explore, which became the focus for the session. This was followed by the induction of an ASC (ca. 12 min), during which the Guide provided an autogenic relaxation induction. The chosen “Nurturing” music program (Bonny, 1980b) lasted around 34 min (see below for details) and was not modified. When the music program was finished, a return phase from the ASC followed, in which the Traveler and the Guide remained silent. After the session, the Guide and Traveler had a post-talk for ca. 6 min.

#### Music

For this session, the Guide chose the original Nurturing program from Bonny created in 1980 (Grocke, 2002). In an interview with D. Grocke, Bonny explained the title and her intentions for this GIM listening program:

“*…we wanted something that was indicative of nurturing of all sorts, not only childhood experiences of nurturing but a close warm feeling, which I feel we have. (…) The Nurturing tape was designed to do just that: to nurture. (…) A lot of it goes back to childhood, their lack of comfort and nurturing, or their perceived lack of it. They have to get beyond their anger and get down to positive feelings.”*

The pieces listened to during the journey are from Britten: *Simple Symphony: Sentimental Sarabande*; Vaughan-Williams: *Prelude on Rhosymedre (4:10 min)*; Berlioz: *L'Enfance du Christ* (Flight into Egypt, Overture (6:55 min); Shepherd's Chorus (5:00 min); Puccini: *Madame Butterfly* (Humming Chorus) (2:47 min); Massenet: *Scenes Alsaciennes* (Sous les Tilleuls) (4:58 min); Canteloube: *Songs of the Auvergne* (Brezairola) (3:13 min).

A recent study by Dukic et al. (2019), testing the GIM process of the “Nurturing” program with 23 participants, confirmed that music of this type can have the psychological function of creating an emotional-scenic background.

### Data Recording and Analyses

#### Content and Video Analysis

A verbal transcript of the video-taped GIM session was annotated with ELAN (Version 5.3, 2018, Max Planck Institute for Psycholinguistics, Nijmegen, The Netherlands). ELAN (Wittenburg et al., 2006) was also used in the event-related analysis of the FAA and PAA (see section “Event-Related Emotional Processing” below). For this, the time series of the FAA values were imported into ELAN and events were located correspondingly.

About 4 weeks after the session, we instructed Traveler, Guide, and two independent raters (see section “Participants”) via email to identify three moments of interest (MOI) based on the video recording of the session, i.e., parts that they think are of special interest and importance as well as a moment that was of no-importance (MONI) for the GIM therapy session. We did not ask for any specific emotional content to be identified but to describe, analyze and reflect upon these moments from a therapeutic perspective. See Supplementary Material for a short video excerpt relating to **Table 3** and **Figure 4**.

**Figure 1:**
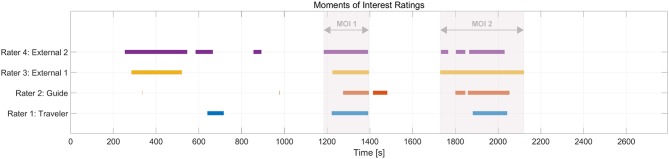
Ratings and overlaps of the Guide-Traveler dyad and 2 independent raters. The red shaded areas show the segments that entered the subsequent analyses (“MOIs”). Note: Timeline displays amounts of seconds related to EEG recording; colors match with Table 1.

Based on the identified individual MOIs from each rater, we identified two overlaps in MOIs across raters; that is, parts of the session all raters evaluated as being interesting (see Figure 1). Based on these overlaps, all further analyses focused on two time periods that ranged from the earliest beginning of an individual MOI included in each overlap to the latest end of an individual MOI included in the same overlap; in other words, ranges of overlaps of individual MOIs (see two red-shaded boxes in Figure 1; see Table 1 for details related to the content). This procedure allowed us to focus on parts of the session that all raters agreed upon while not having to analyze only parts of individual MOIs. For reasons of simplicity, henceforth, we refer to these ranges of overlaps as MOI 1 and MOI 2 (in contrast to individual MOIs, identified by the raters). The duration of MOI 1 and MOI 2 were 3.31 min and 6.31 min, respectively. When necessary, parts of MOIs will be referred to as segments of MOIs. The duration of the MONI was 3.32 min.

**Table 1:** Detailed MOI selections, descriptions and themes of the session.

**Person**	**Start**	**End**	**MOI description - Statements**	**MOI Overlaps - Extract**
Rater 4	13:53:09	13:58	stable (touching the wood) – new branches of my own tree – many roots – pregnancy – birth – traumatic events- nobody there – needed “hand on my head”	
Rater 3	13.53.40	13.57.35	In this darker, minor section of the music the image of the TREE is developed, and related to memories of childbirths and difficult feelings/family traumas.	
Traveler	13.59.35	14.00.51	Connect to the female roots. Music bringing energy and light.	
Rater 4	13:58:40	14:00	A message “I am with you” and my mother told me “I am with you” – music	
Rater 4	14:03:11	14:03:47	A dream of looking at her (unborn) son. A message from him “don't worry - I went up - to the breast cave so I am safe”	
Guide	14:05:12	14:05:12	Empowerment of the women in the roots of her family	
Rater 4	14:08:40	14:09:52	Not recognized by mother because being pregnant without being married “Couldn't you have it removed?”.	MOI 1 - SEGMENT 1
				1) Negative emotion 2) Positive emotion emerging image of the **Grandmother** 3) Message from Grandmother
Traveler	14.09.17	14.12.07	Grandmother singing. Being told not to worry. Music coming into the body and Heart. Experienced from inside as a pivotal moment	
Rater 3	14.09.20	14.12.10	Grandmother sends a message. Music for the body and the heart (long sequence without talking).	
Rater 4	14:09:52	14:12:06	The music – really coming into her body: “Coming into my heart”	MOI 1 - Segment 2
				Positive emotion: Music fills her heart
Guide	14:10:10	14:12:10	Music coming into her heart	
Guide	14:12:30	14:13:36	Client sees the green light around her daughter in Law (strong visual image)	
Rater 3	14.17.45	14.22.45	“Music is nurturing” - Connecting to unborn baby and wishing all the best.	MOI2 - SEGMENT 1
				1) Music is nurturing 2) A **message to the baby**
Rater 4	14:17:48	14:18:21	Music is very nurturing.	
Guide	14:18:56	14:19:41	Gives message to baby	
Rater 4	14:18:58	14:19:41	Feel connected to the unborn baby of her son. Says:” Welcome. hope you'll have a nice life”.	
Guide	14:19:54	14:23:08	Brezairola (strong transference to the female voice)	MOI2 - SEGMENT 2
				1) **Lullaby** for the baby 2) In contact 3) Family roots
Rater 4	14:20:00	14:21:00	the music is “Singing is for the baby”	
Traveler	14.20.17	14.22.57	The tree is singing. From inside the tree and the roots. Embracing the tree with my Family hand in hand	
Rater 4	14:21:00	14:22:45	Hand in hand around the tree. The tree is singing – from inside –roots	
Rater 3	14.22.45	14.24.15	Therapist summarizes the session with focus on the TREE experience. Very close contact.	

*Colors indicate raters (corresponding with Figure 1) and overlap; time refers to the actual time of day of the recording, comments/statements are entered subsequently, according to their start time*.

The analysis of these MOIs followed a micro-analytic approach (Wosch and Wigran, 2007): the therapy process was submitted to a moment-to-moment analysis, in combination to the parallel analysis of the EEG (see below).

#### EEG

The EEG was recorded with 500 Hz from 32 Ag/AgCl active electrodes (ActiCap, Brain Products GmbH, Germany) for each participant, placed according to the extended 10-20 system (FP1, FP2, F7, F8, F3, F4, Fz, FT9, FT10, FC5, FC6, FC1, FC2, T7, T8, C3, C4, TP9, TP10, CP5, CP6, CP1, CP2, Pz, P7, P8, P4, P3, Oz, O1, O2). The ground electrodes were placed on Fpz on each participant, and were recorded by using a ground distributor connected to an ActiCHamp amplifier (Brainproducts GmbH, Germany). This amplifier implements a reference-free design, i.e., there is no dedicated hardware reference electrode and a reference electrode (Cz of participant 1) was only chosen in the recording software (Brainvision Recorder, version 1.21.030, Brain Products GmbH). As only a single reference electrode can be chosen there, the first offline pre-processing step involved re-referencing the EEG signals of participant 2 to Cz of participant 2, thereby eliminating the influence of the online shared reference on the data.

Simultaneous video and audio was recorded with a Sony HVR Z1E camera, connected via Firewire to the EEG-recording PC. EEG and video signals were synchronized with the BrainVision Video Recorder (Brain Products GmbH, Germany).

Data were offline first analyzed with the BrainVision Analyzer software (version 2.1.2; Brain Products GmbH). An Independent Component Analysis was performed (Infomax extended) and components reflecting horizontal and vertical eye movements and blinks were subtracted from the data. Data were then filtered with a 4th order Butterworth IIR filter (1–40 Hz; 50 Hz notch).

Due to a poor scalp-electrode contact during recording, four electrodes were found - based on visual inspection - to show large amounts of noise (mostly in the 50 Hz line noise range, but in lower frequency ranges as well as). Therefore, electrodes O2, P8 and TP10 of the Guide and FP1 of the Traveler were interpolated by spherical (4th order) splines.

After computing current source densities (CSD) from the scalp-recorded voltages, data were divided into 1 s epochs with 0.5 s overlap and artifactual epochs were automatically detected and manually checked (based on scalp-recorded voltage fluctuations). Automatic rejection criteria were: maximal 50 μV voltage step, maximal allowed difference of 200 μV in 200 ms intervals, absolute threshold of ± 100 μV, and lowest activity of 0.5 μV in 100 ms intervals. A Fast Fourier Transformation (FFT) was performed (max. resolution 0.977 Hz) on all artifact-free epochs, which were Hanning windowed (10%).

Power values for all Fourier coefficients were exported and further analyzed in Matlab (version 9.2.0, Mathworks, Inc.). Alpha power for each segment was calculated as the band (8–13 Hz) value sum and the alpha asymmetry scores were computed by taking the differences between the natural-log transformed power values of frontal electrodes F4 and F3, and between P3 and P4. In addition, power in the theta (4–8 Hz) frequency band was calculated for electrode Fz.

For investigating the time course of alpha asymmetries, missing values (due to artifactual epochs) were linearly interpolated and the time series were filtered (Savitzky-Golay, 3rd order, 11 frames). In addition, mean alpha power across different conditions (rest, moments of interest, moment of no interest) was calculated for an occipital region of interest (ROI) with electrodes O2, Oz, and O1. For EEG source localization, data were re-referenced to average reference and submitted to a Low Resolution Electromagnetic Tomography (Pascual-Marqui et al., 1994) analysis as implemented in BrainVision Analyzer.

Results of occipital alpha power and frontal alpha asymmetry during resting-state and moments of (no) interest were statistically analyzed with paired samples *t*-tests in SPSS using case-by-case exclusion for missing values (Version 24; IBM, USA). *P* values were Bonferroni corrected by multiplying the observed *p*-values with 9 (for FAA in the Traveler) or 6 (FAA for Guide, PAA, FMT, occipital alpha power). Cross-correlations were computed in R (R Development Core Team, 2008).

## Results

### General Comments on the GIM Session

The external GIM therapists examined the *overall content and range of core topics* of the therapy session as:

External rater 1: “A very authentic and moving session; The travel is a mixture of memories—body experiences—core imagery—and even a message; guiding is sparse, mostly connecting imagery to body and emotions.”

External rater 2: “The images in the journey are rich: colors, memories, feelings, body sensations, spiritual experiences (feeling a presence very close—her boy), dialogue and messages. Themes are trees, roots, family history, females and giving birth (reproduction), love. Enclosed are images/messages that stood out.”

The Guide stated: “I am aware that the analysis could focus on different types of experiences (visual, memories, embodied imagery, transpersonal, and transference to the music). I was also aware of moments when I felt very close to the Traveler's experiences, and for want of a better word I've called those moments “mutuality.” I felt almost a leap in my heart when she mentions that her mother experienced the same problem, realizing the generational impact of it. I also felt very close to the Traveler's experience during the Berlioz Chorus—the 3rd verse of the chorus where there is a strong crescendo in the music. Another point was during the whole of the Brezairola—[the Traveler] has a strong transference to the female voice, and I am silent so I don't interfere with it. But I felt very connected with her for the duration of that piece.”

The Traveler was also asked for her experiences. She described her experience of parts of the session as “pivotal” and which will be reported in more detail below.

### Moments of Interest and Imagery

As can be seen in Figure 1, all raters identified moments of interest in the second half of the session that clearly overlapped with each other. All subsequent analyses will focus around these overlaps (red-shaded area in Figure 1, henceforth just called “MOIs,” see section “Content and Video Analysis”) and smaller segments within these MOIs. Table 1 lists the corresponding detailed MOI selections, descriptions and themes of the session returned from Traveler and Guide, and from the independent raters.

In contrast to the above-mentioned moments, the moment of no-interest (MONI) was from the beginning of the session (13:49:15–13:52:47) and marks the exploration in the starting phase of the journey in which particular colors are imagined and the imagery has not yet developed a particular focus. All raters agreed that it was not easy to select MONIs.

### Differences in Imagery-Related Activity

**Figure 2:**
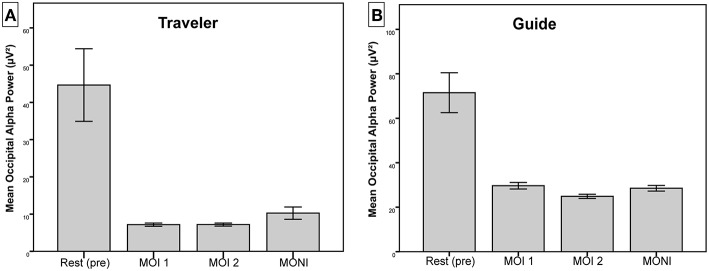
Mean alpha power over occipital ROI for **(A)** Traveler and **(B)** Guide. Error bars represent ± 2 standard error of the mean.

Figure 2 shows averaged occipital alpha power during the resting-state, the two overlapping segments of important moments, and during the non-important moment for the Guide and the Traveler. For both participants, alpha power was larger during the resting-state compared to therapeutically interesting moments and compared to the non-interesting moment. In the Traveler, alpha power was higher during the moment of no interest compared to both interesting moments, which in turn did not differ in terms of alpha activity. In the Guide, alpha power differed between the two important moments and between MOI 2 and MONI (for statistical results, see Table 2).

**Table 2:** Summary of paired sample *t*-tests for occipital alpha power during rest, important and non-important moments for the Traveler and the Guide.

		***t*-value**	**Degrees of freedom**	***p*-value**
**TRAVELER**				
Rest vs.	MOI 1	7.69	381	0.0006
Rest vs.	MOI 2	7.92	452	0.0006
Rest vs.	MONI	6.88	395	0.0006
MOI 1 vs.	MOI 2	−0.064	381	0.949
MOI 1 vs.	MONI	−3.62	381	0.0006
MOI 2 vs.	MONI	−3.68	395	0.0006
**GUIDE**				
Rest vs.	MOI 1	9.71	400	0.0006
Rest vs.	MOI 2	12.19	443	0.0006
Rest vs.	MONI	9.37	388	0.0006
MOI 1 vs.	MOI 2	5.23	400	0.0006
MOI 1 vs.	MONI	1.22	388	0.224
MOI 2 vs.	MONI	−4.6	388	<0.0006

*MOI, Moment of interest; MONI, moment of no interest*.

These findings suggest increased activity in the visual cortex during important moments compared to the resting-state for both participants, as well as increased activity in visual areas during important compared to non-important moments in the Traveler.

**Figure 3:**
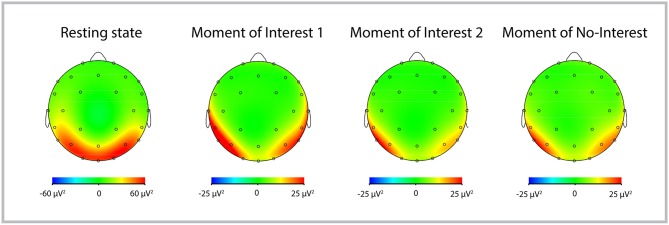
Topographic distribution of alpha power of the Traveler during Rest, MOI 1, MOI 2, and MONI. Red colors represent more alpha power, indicating less cortical activity.

As shown in Figure 3, this difference in power is likely due to a shift of the topographical distribution of alpha power. Whereas during the resting-state, alpha power showed a predominantly occipital distribution, there appeared to be less occipital power and a change to a more temporo-parietal power distribution during MONI.

### Differences in Emotion-Related Activity

#### Frontal Alpha Asymmetry

The frontal alpha asymmetry of the *Traveler* was more positive (indicating positive emotional processing) during therapeutically important moments (MOI 1: *M* = 0.13, *SD* = 0.7; MOI 2: *M* = 0.06, *SD* = 0.74) compared to the resting-state (Rest1) prior to the session (*M* = −0.17, *SD* = 0.75; Paired sample *t* tests Rest1 vs. MOI 1: *t*(361) = −5.6, *p* < 0.0009; Rest1 vs. MOI 2: *t*_(421)_ = −5.14, *p* < 0.0009). Further, the FAA was more positive during the moment of no interest (*M* = 0.84, *SD* = 0.73) compared to both moments of interest (*t*_(359)_ = −13.12, *p* < 0.0009; *t*_(365)_ = −14.21, *p* < 0.0009). There were no differences in FAA between the two different MOIs (*t*_(354)_ = 1.5, *p* = 0.14). There were no differences between the resting-state prior and after the session (*t*_(428)_ = −0.49, *p* = 0.62).

In contrast, the FAA of the *Guide* did not differ between the Rest1 (*M* = 0.05, *SD* = 0.65) and the first important moment (*M* = −0.02, *SD* = 0.65); paired sample *t* tests Rest1 vs. MOI 1: *t*_(393)_ = 1.56, *p* = 0.119. However, the FAA was more negative during the second important moment (*M* = −0.3, *SD* = 0.69) than during the resting-state (paired samples *t*-test Rest1 vs. MOI 2: *t*_(389)_ = 7.35, *p* < 0.0006). Further, the FAA was more positive during the moment of no interest (*M* = 0.16, *SD* = 0.57) compared to both moments of interest (*t*_(371)_ = −4.58, *p* < 0.0006 and *t*_(333)_ = −9.24, *p* < 0.0006, respectively). Additionally, the second MOI showed a more negative FAA compared to the first MOI (*t*_(351)_ = 5.51, *p* < 0.0006). There was no difference (*t*_(437)_ = 2.32, *p* = 0.16) between pre and post resting-states.

#### Parietal Alpha Asymmetry

The parietal alpha asymmetry (PAA) of the *Traveler* was more positive during the Rest1 (*M* = 0.095, *SD* = 0.58) compared to the first, but did not differ compared to the second MOI (MOI 1: *M* = −0.031, *SD* = 0.49; MOI 2: *M* = 0.015, *SD* = 0.52: paired sample *t* test Rest1 vs. MOI 1: *t*_(361)_ = 3.18, *p* = 0.012; Rest1 vs. MOI 2: *t*_(421)_ = 1.85, *p* = 0.39). There were no differences in PAA between the two MOIs, nor between the interesting and non-interesting moments (all *p*'s > 0.103). The PAA was significantly more negative during Rest2 compared to Rest1 (*t*_(428)_ = 4.279, *p* < 0.0006).

The PAA of the *Guide* was more positive during the Rest1 (*M* = 0.229, *SD* = 0.823) compared to both MOIs (MOI 1: *M* = −0.53, *SD* = 0.69; MOI 2: *M* = −0.72, *SD* = 0.64: paired sample *t*-test Rest1 vs. MOI 1: *t*_(393)_ = 13.86, *p* < 0.0006; Rest1 vs. MOI 2: *t*_(389)_ = 19.19, *p* < 0.0006). Additionally, the PAA was more negative during the second MOI compared to the first one (*t*_(351)_ = 3.17, *p* = 0.012), as well as compared to the moment of no-interest (*t*_(333)_ = −3.191, *p* = 0.012). There was no difference in PAA during the first MOI compared to the moment of no-interest (*M* = −0.57, *SD* = 0.67; *t*_(371)_ = 0.93, *p* = 0.35), and there was no difference between the pre- and post-resting-state (*p* = 0.952).

#### Frontal Midline Theta

For the *Traveler*, frontal midline theta power (FMT) was increased during the resting-state (*M* = 43.01, *SD* = 28.91) compared to both MOIs (MOI 1: *M* = 2.81, *SD* = 2.345; MOI 2: *M* = 2.7, *SD* = 1.56; rest vs. MOI 1: *t*_(361)_ = 26.13, *p* < 0.0006; rest vs. MOI 2: *t*_(421)_ = 27.47, *p* < 0.0006). Compared to MOI 1 and 2, FMT was increased during the moment of no-interest (*M* = 11.33, *SD* = 6.71; MONI vs. MOI 1: *t*_(359)_ = −22.78, *p* < 0.0006; MONI vs. MOI 2: *t*_(365)_ = −24.28, *p* < 0.0006). FMT did not differ between both MOIs (*p* = 0.63). FMT was higher in the resting-state before the session compared to after the session (*t*_(428)_ = 14.05, *p* < 0.0006).

Similarly, frontal midline theta power (FMT) of the *Guide* was also increased during the resting-state (*M* = 101.21, *SD* = 54.45) compared to both MOIs (MOI 1: *M* = 4.63, *SD* = 2.21; MOI 2: *M* = 5.27, *SD* = 2.78; rest vs. MOI 1: *t*_(393)_ = 35.19, *p* < 0.0006; rest vs. MOI 2: *t*_(389)_ = 35.26, *p* < 0.0006). The FMT during the moment of no-interest (*M* = 5.15, *SD* = 2.56) was increased compared to MOI 1 (*t*_(371)_ = −2.72, *p* = 0.042), but did not differ compared to MOI 2 (*t*_(333)_ = 0.04, *p* = 0.97). In addition, MOI 2 showed larger FMT values than MOI 1 (*t*_(351)_ = −3.42, *p* = 0.006). Similar to the findings of the Traveler, FMT was higher in the resting-state before the session compared to after the session (*t*_(437)_ = 19.63, *p* < 0.006).

### Event-Related Emotional Processing

#### MOI 1: Pivotal Moment—Message From Grandma

Here we are looking at two interrelated segments (see Tables 3, 4). The first describes a “message from the grandma” and the second is a description how the “music is filling up her heart.” We asked the Traveler to expand on her experiences in more detail in order to contextualize the data accordingly. The Traveler described this MOI as a pivotal moment. Before this segment she spoke about negative emotions relating to her son's birth. Her mother was critical of her because she was pregnant without being married. “Couldn't you have it removed?” she had asked, and the Traveler felt very angry. But despite this her parents had been very loving and caring toward her son. After she told this, she listened to the music (2nd verse of Shepherd's Farewell to the Holy Family—Berlioz' L'enfance du Christ) and then reported after the imagery that she just had received a message from her grandma, who always had a positive influence on her. Seeing her grandmother telling her “not to worry,” was a pivotal moment (see Table 3 below and Supplementary Material for a short video excerpt of this segment).

**Table 3:** MOI 1 segment1, pivotal imagery, transcript of Traveler and Guide and event-related frontal alpha asymmetry (FAA) peak values.

**MOI 1, Segment 1**	**Transcript (Commentary)**		**FAA peaks**	
**Time**	**Traveler**	**Guide**	**Traveler**	**Guide**
14:09:00	“…. But when I was pregnant, they were very, very careful, very, they'd loved [NAME OMITTED], both of them.		+1.2099	
14:09:08	Music listening			
14:09:11	(emerging imagery of the Grandma?)		−0.8400	
14:09:15	Opens mouth			
14:09:16			+0.2729	
14:09:17	Oh, my love, that's too much beauty			
14:09:20				+0.5270
14:09:21		yeah?		
14:09:23	I feel like something is coming from my grandmother (seeing my grandmother flying towards me with her long gray hair and looking into my eyes with care)		+0.7910 +0.7259	
14:09:27		yes?!		
14:09:30				+0.7980
14:09:35	Like she is telling me not to worry so much …		+1.8240	
14:09:39	Music listening			

*Underlined words correspond with peaks. FAA peaks are log-transformed (ln) power differences of relative AA (here ln F4 - ln F3). For example, positive values (like +1.8240) indicate relative higher activity in the left-frontal cortex during the corresponding time segment (14:09:35)*.

**Table 4:** MOI 1, segment 2; pivotal imagery, transcript of Traveler and Guide and event-related frontal alpha asymmetry (FAA) peak values.

**MOI 1, Segment 2**	**Transcript (Commentary)**		**FAA peaks**	
**Time**	**Traveler**	**Guide**	**Traveler**	**Guide**
14:09:51				+0.8220
14:09:53			+0.7490	
14:09:57	Like the music is … really		+1.0839	
14:10:07	…Coming into the body…		+1.12	
14:10:10		(adjusts volume on iPod)		−1.7379
14:10:15	… feeling it…		+0.4610	
14:10:25	… and, coming into the heart			
14:10:27				+0.5766
14:10:29	Music listening		−0.6609	
14:10:32				+0.6490
14:10:35		Take in as much as you want		
14:10:41	Music listening		+1.1450	

*Underlined words correspond with peaks. FAA peaks are log-transformed (ln) power differences of relative AA (here ln F4 - ln F3). For example, positive values (like +1.1450) indicate relative higher activity in the left-frontal cortex during the corresponding time segment (14:10:41)*.

Written comment from Traveler:

“*Like the music is becoming the spirit of my grandmother and the voices bring the message. I feel relieved and feels as if the music gets into my body and I feel some lightness in the body and at the same time feel spiritually connected to my grandmother. Suddenly it feels like my heart is opening towards the music and the music and the spiritual sound is filling my heart – thus experiencing my heart much bigger and warm and thankful. I am touched and get into tears. It feels very relieving. I feel like being very peaceful.”*

We will now look how this moment unfolds along the conversation and the parallel peaks of the FAA representing the continuous emotional processing. When peaks coincided with spoken words they are underlined in the table. In the description of the two segments of MOI 1 we can study the interpunction of emotional processing in the Traveler and in the Guide.

##### MOI 1 - Segment 1

The first positive peak corresponds with talking about the Traveler's parents' loving care for her son (after telling that her mother was first suggesting to abort). This is followed by section in which the pivotal image of the grandmother probably emerges, interestingly accompanied with a negative peak. Then she tells about her emergent imagery and successive positive peaks culminate in the pivotal message sent from her grandma “not to worry.”

**Figure 4:**
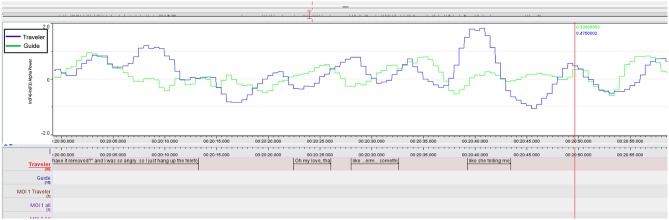
MOI 1, Segment 1. Time course of frontal alpha asymmetry (F3/4, 0.5 s bins), indicating interplay of emotional processing of Guide (Green) and Traveler (Blue). For a description of event-related peaks, see Table 3.

Inspecting the emotional contour of both we can see how the peaks of the Guide (see Table 3 and Figure 4) increase in parallel, especially when she recognizes that there is an important imagery emerging, signified with the Traveler's words: “oh, my love …,” but culminate a second later than the Traveler's FAA peaks (See Supplementary Material for a short video excerpt).

##### MOI 1 - Segment 2

**Figure 5:**
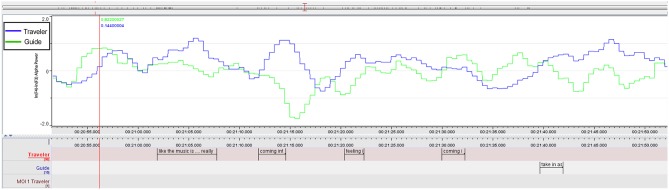
MOI 1, Segment 2. Time course of frontal alpha asymmetry (F3/4, 0.5 s bins), indicating interplay of emotional processing of Guide (Green) and Traveler (Blue). For a description of event-related peaks, see Table 4.

Both share this moment by knowing about the importance of this message and, while the negative peak of the Guide relates to adjusting loudness on the iPod, her next two peaks (14:10:32) mark the advice to the Traveler to “take in as much as you like.” This leads into a longer part, in which the Traveler enjoys the deep relief until the end of this “woman's choir” (Berlioz: L'Enfance du Christ, 3rd verse of Shepherd's Farewell), as the Traveler describes it. Results of the FAA cross-correlation between Traveler and Guide during this segment (Figure 5 and Table 4) are reported below.

For completeness here are the lyrics of the 3rd verse sung by the choir:

Dearest infant, may God bless you;

*God bless your happy parents too*.

May injustice never darken

*the love that God bestows on you*.

*Guardian angels guide, protect you*.

*May they lead you safely through!*^1^

All raters overlapped from 14:10:10–14:12:06 were agreeing that the part in which the music fills the heart and a woman's choir accompanying the relief constituted an important MOI.

#### MOI 2: Mutuality

##### MOI 2-Segment 1

The second MOI that all raters agreed upon and the range of ratings covered (see Figure 1 above) was described and annotated (see also Table 5 below) from the Traveler as follows:

T: ‘The singing is for the baby'

COMMENT T: (feeling like being connected to the unborn baby through the music– like me singing to the baby. Feel the three (husband, son and daughter in law around me) –

T: ‘…we are all standing hand in hand around the tree…'

COMMENT T: A feeling like the tree is singing from inside – from inside the roots- like a very strong power – giving a deep feeling of grounding and really connected to the tree and the roots and my family. Like all of us are filled out and grounded and connected by this strong and spiritual voice. A feeling of power.”

The following two segments of MOI 2 were annotated with the corresponding FAA and PAA peaks.

**Table 5:** MOI 2 Segment 1. Transcript of Traveler and Guide and event-related alpha asymmetry peak values.

**MOI 2, Segment 1**	**Transcript (Commentary)**		**FAA and PAA peaks**	
**Time**	**Traveler**	**Guide**	**Traveler**	**Guide**
	…Like the music is very nurturing…			
14:17:48	listening		+0.6212	
14:18:08	Sadly it is also containing the sad thing about it, don't know…in a nurturing way		+0.9589	
14:18:30	Exhales deeply		P3-4: +0.8598 F4-3: −0.8358	
14:18:53	I feel like almost being connected to the baby		+0.1879	
	Well I know, It is very smart			
14:19:03	listening		+1.0179	

*Underlined words correspond with peaks. FAA peaks are log-transformed (ln) power differences of relative AA (here ln F4—ln F3 for FAA or ln P3—ln P4 for PAA). For example, positive values (like +1.0179) indicate relative higher activity in the left-frontal cortex during the corresponding time segment (14:19:03); unlabelled values represent FAA asymmetry scores. P, Parietal; F, Frontal Asymmetries*.

##### MOI 2-Segment 2

In the following segment the therapist asked the Traveler, after connecting to the unborn baby, (see Table 6) if the Traveler wants to send something “to the baby.” With the second sending the voice of the female singer started into another verse of Canteloube's “Brezairola” (a lullaby) and here the Guide “felt very close” to the Traveler, signifying this part as a strong “moment of mutuality” (see above).

**Table 6:** MOI 2 segment 2. Transcript of Traveler and Guide and event related alpha asymmetry peak values.

**MOI2, Segment 2**	**Transcript (Commentary)**		**FAA and PAA peaks**	
**Time**	**Traveler**	**Guide**	**Traveler**	**Guide**
14:19:11		Is there something you want to say to this baby?		+0.2520
14:19:18	listening		P3-4: +0.7393	
14:19:19	Welcome!		P3-4: +0.7060	
14:19:25	Hope you will have a nice 9 month!		+0.7729	
14:19:20 - 54	Traveler and Guide ‘feeling of mutuality' (see Figure 5)			
14:19:50		Is there something you can send to this baby?		F3-4: +0.3779 P3-4: +0.3129
14:19:57	Yeah, a lot of, good energy, hope, love		P3-4: +0.8945 F4-3: +0.8009	
14:20:14	listening		+0.6879	
14:20:20	The singing is for the baby		+0.6300	
14:20:59	Like we all are			
14:21:03	listening			+0.8257
14:21:07	all 4 standing around, hand in hand around the tree, and the tree is singing from inside, from inside he answers us, the roots		+1.0937	

*Underlined words correspond with peaks. FAA peaks are log-transformed power (ln) differences of relative AA (here ln F4—ln F3 for FAA or ln P3—ln P4 for PAA). For example, positive values (like +1.0937) indicate relative higher activity in the left-frontal cortex during the corresponding time segment (14:21:07); unlabelled values represent FAA asymmetry scores. P, Parietal; F, Frontal Asymmetries*.

**Figure 6:**
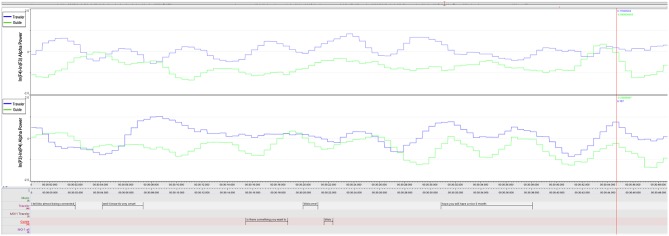
MOI 2, Segment 1. Time course of frontal and parietal alpha asymmetry (F3/4, P3/4, 0.5 s bins), indicating interplay of emotional processing of Guide (Green) and Traveler (Blue). For a description of event-related peaks, see Table 5.

We can trace this mutuality graphically in the plot of the F3/4 FAA dynamics in Figure 6 and in the cross-correlation reported in the next paragraph. Both ELAN screenshots contextualize Traveler and Guide's verbalizations of guidance and imagery, and also the asymmetry data inspected along the time course of therapy.

#### FAA Cross-Correlation During MOI 1 and 2

Based on the comments of the Guide about her feeling very close to the experiences of the Traveler (see section “General comments on the GIM Session” above), we further explored a possible relationship between the emotional processing in the Traveler and Guide. For that, we calculated the cross-correlation between the FAA time series during two segments of MOI 1 (Berlioz piece: 2.18 min, 14:09:52–14:12:10) and MOI 2 (Brezairola piece, 3.14 min, 14:19:54–14:23:08) that were explicitly highlighted by the Guide (see **Figure 8**). Results showed a significant cross-correlation during the first MOI at lag 5 (*p* = 0.031; cross-correlation coefficient = 0.138), as well as significant cross-correlations for lag 0 (*p* = 0.011) and −1 (*p* = 0.001) during the second MOI (cross-correlation coefficients 0.138 and 0.178 for lag −1 and 0, respectively).

#### LORETA Analysis of Emerging Imagery

The highest peak in FAA in the Traveler occurred in the first segment of MOI 1, when the Traveler expressed that her Grandma is telling her not to worry. Thus, it is likely that this part of the session is of pivotal importance. The imagery may have emerged from 14:09:08 to 14:09:17, prior to the verbalization of the imagery (see Table 4). To further explore brain activity during this particular segment, we submitted the time-domain EEG (14:09:12-17) to a source localization analysis with LORETA (see Figure 9). Results showed a best match at 3 mm in BA 39 within the posterior parts of the Middle Temporal Gyrus (*X* = 52, *Y* = −67, *Z* = 8). A comparison with a segment of the same length (5 s) during the moment of no-interest during which an image of a color was processed showed a peak activation at the same cortical areas. However, the strength of the activation during the MOI was nominally clearly higher than during the moment of no-interest (0.00106 μA mm^2^ vs. 0.000255 μA mm^2^, respectively).

#### FAA Difference Between Induction of Altered State and Rest

The FAA in the Traveler was more negative during the induction of the altered state of consciousness (*M* = −0.91, *SD* = 0.77) compared to the resting-state (*M* = −0.208, *SD* = 0.71; *t*_(372)_ = −13.24, *p* < 0.0009), as well as compared to both moments of interest (all *p*'s < 0.0009).

## Discussion

In this first Hyperscanning recording of a music therapy session, we were investigating the natural GIM session flow and investigated the continuous time series of a dual-EEG of a Traveler (client) and a Guide (therapist). This provided a dataset with high ecological validity, including an authentic therapeutic relationship between a Traveler and a Guide.

The analysis of session ratings showed overlaps between all raters, confirming the importance of two particular shared moments of interest (MOIs) in the session and that these moments are of distinct content from moments with no-importance (MONI). The range and the particular overlaps (see Figure 1) offered a reliable base to select relevant material to further study the experienced imagery as it emerged in more detail.

For the exploration of imagery processing, we used occipital alpha power as an index of activity in cortical visual areas. For the exploration of emotional processing, several central markers of emotion were used, namely the frontal and parietal alpha asymmetry and frontal midline theta power. These markers were used (a) to investigate general differences between the resting-state and therapeutically important and non-important moments and (b) to explore the time course of emotional processing and a possible relationship between Traveler and Guide during the session. For this objectivist case study (see Ridder and Fachner, 2016) we formulated a few propositions (see end of “Aims” section) and will discuss the corresponding results.

### Moment of Interest 1: Message From Grandma

*Proposition V: We expect strong peaks to occur within imagery events of strong emotional valence*.

In the first MOI, an important personal visual imagery and a corresponding dialogue with an imagined significant person constituted a pivotal moment. In this moment, the Traveler received a message from her grandma, which was accompanied by a negative peak in FAA (see Table 3) indicating a small sequence of negative emotion processing; however, verbalizing the message to the Guide, and here especially the meaning—“telling me not to worry”—, showed the largest positive FAA peak in that sequence (see Figure 3) and this confirmed our proposition V. It is likely that the Traveler immediately recognized the positive impact of the message, but that the negative emotional impact of the previous narrative about being asked to abort (that was still present when the message occurred) transformed into a positive emotion when being told “not to worry” (see Table 3). This all happens during a passage in the music in which the choir sings blessings to parents and a child (see results section “MOI 1–Segment 1”). For both Traveler and Guide, being experienced GIM therapists themselves, this coincidence of the message and the music may have added to the emotional significance this moment has on several layers.

In previous electrophysiological and imaging studies, a right hemispheric MTG asymmetry has been associated with face recognition (Ojemann et al., 1992; Gur et al., 1993). In particular attractiveness of faces (Vartanian et al., 2013) seems to be processed in the right MTG. In a recent functional magnetic resonance imaging study on negative emotion in GIM, Lee et al. (2016) reported stronger peak intensity in the left MTG when comparing a GIM condition with a condition that included only a verbal instruction, whereas the comparison between GIM and only music listening showed no lateralization effects. Compared to that study, our source localization results (see Figure 9) showed a more posteriorly peak MTG activation. One might speculate that a verbal instruction as heard from the Traveler's Grandma (“not to worry”) may also be processed in the left MTG.

However, recent investigations into Social Anxiety Disorder reported impaired activity in the left MTG (Yun et al., 2017). Given the significance of the message delivered from the Grandma in terms of the change that was brought forward in the therapy, the moment in which the image appeared may have been accompanied with the general fear and anxiousness of losing the child, which in turn fits with the observation of reduced overall FMT values and less positive PAA shifts during MOI 1.

For completeness it has to be reported that the FAA displayed a negative peak, and that the Traveler was swallowing during that moment^2^. Because swallowing rates have been shown to be modulated by affective states (Cuevas et al., 1995; Ritz and Thöns, 2006), this reaction might also point to the strong emotional impact of the emerging image that had to be “digested” here. Nevertheless, after the image of the Grandma appeared, a successive increase of positive FAA peaks appeared (Table 3) culminating in the verbal report of the Traveler that the Grandma told her “not to worry too much.”

#### Shared Temporal Dynamics of Emotion

*Proposition VI: We expect the peaks to indicate a temporal dynamic of dyadic biosemiotic interpunction of the therapy process, i.e., peaks indicating shared emotional processing*.

Schall et al. (2015) pointed out that music therapy action can be traced in the time series of therapy events and reported corresponding decreases and increases of situational well-being and emotional expression during different phases of the intervention. In our study, in Tables 3–6, we can see how the emotional contour of the session unfolds and how the biosemiotic interpunction regarding emotional processing aligns between Guide and Traveler, thus confirming proposition VI.

Thus, by inspecting the AA peaks, we can trace the emotional intensity occurring during both, verbal and non-verbal events, for example when the Traveler reported the message but also when the Traveler was only listening/imagining without any verbal utterances (14:10:41). It also showed how the situated emotional processing builds up to culminate in a positive peak when the reassuring message of the grandma was verbalized. Here the culminating peaks may indicate a situated cognitive process in which the Traveler gains insight into her own family roots and history of childbirth and ‘emotions signifying meaning are evoked through the interplay of music and the people' (Fachner, 2014, p 793); in this case with Berlioz's music and lyrics (see section “MOI 1–Segment 2” above) as a soundtrack to the Traveler's inner journey. Situated cognition refers to the inseparability of embodied perception and action and that meanings of the experiences are cognitively processed according to the affordances of the social and physical context in which we are situated (Iyer, 2002; Schwarz, 2002); in this case, the context-related insight that is based on the successive emotional peaks and meaning of the message shared with the Guide. Grandma's message is perceived and recognized as important by both subsequently and shared in an interpunction of increasing peaks.

**Figure 7:**
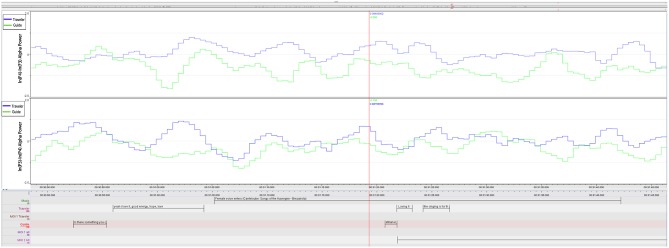
MOI 2, Segment 2. Time course of frontal and parietal alpha asymmetry (F3/4, P3/4, 0.5 s bins), indicating interplay of emotional processing of Guide (Green) and Traveler (Blue). For a description of event-related peaks, see Table 6.

**Figure 8:**
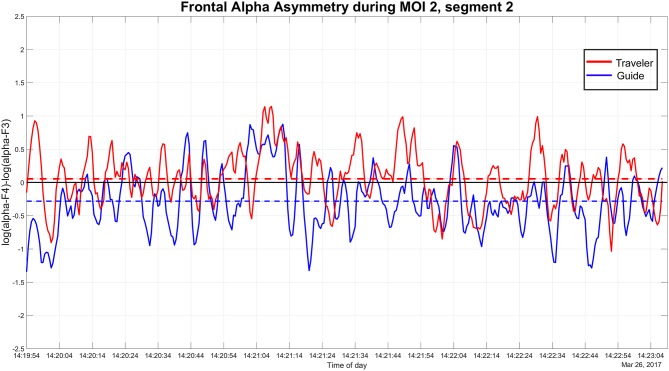
MOI 2, Extended Segment 2. Time course of frontal alpha asymmetry at electrode F3/F4 of Guide and Traveler that entered cross-correlation analysis.

The FAA peaks of the Guide and the Traveler appeared to be aligned during both MOIs (inspect Figures 5–7). Especially during the second segment of the second MOI, which has been described by a “strong feeling of mutuality” by the Guide (see section “General Comments on the GIM Session”), a close alignment of both FAA peaks can be observed (see Figures 6, 7; Table 6). This is also indexed by a significant cross-correlation between the two FAA time series (see Figure 8), perhaps suggesting that shared temporal dynamics of emotional processing is represented in the FAA. Here the Guide described that the Traveler had a “strong transference to the female voice and I am silent so I don't interfere with it. But I felt very connected with her for the duration of that piece.”

**Figure 9:**
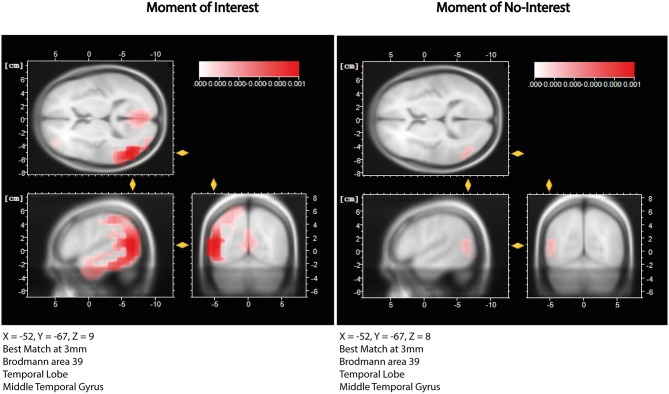
LORETA source localization results. The left panel shows localization estimates for a 5s segment of MOI 1, While the right panel shows estimates for a 5s segment of MONI. In both conditions, the peak activation was estimated to occur in the posterior part of the Middle Temporal Gyrus (BA 39), but was less pronounced during the MONI.

### Differences in Cortical Activity During Rest and Moments of (no) Interest in GIM

During a real-world GIM session, a multitude of different processes are co-occurring. To ensure that our observations reflect imagery- and emotion-related activity, we tested if there are differences in cortical activity in areas known to be involved during imagery and emotion.

#### Imagery: Occipital and Temporo-Parietal Differences

*Proposition III: We expect the visual cortex to be more active (to exhibit less alpha power) during the imagery process than during the resting-state*.

*Proposition IV: Additionally, during GIM (compared to rest), we expect that temporo-parietal areas are less active (i.e. more alpha power) then occipital areas*.

Confirming proposition III and in line with other imagery research (Hunt, 2011; Schaefer et al., 2011) our results showed that cortical activity with regards to occipital alpha power differed between the resting-state and the therapy session (both conditions with no external visual input for the Traveler). Because previous studies have shown the involvement of visual areas in the occipital cortex during music-related imagery (visual and musical; Hunt, 2011; Schaefer et al., 2011), our results of increased alpha power during the resting-state (i.e., less activity) suggest that occipital areas are more activated during the GIM session and hence imagery processes might have been more pronounced.

Furthermore, inspection of the topographical distribution of alpha power suggests that temporo-parietal areas became more inhibited during the therapy, confirming proposition IV. Interestingly, alpha power increases over temporo-parietal regions has been associated with creative-related demands and creative ideation, and could reflect more internally oriented attention (Benedek et al., 2014; Fink and Benedek, 2014). Thus, one might speculate that increased temporo-parietal alpha activity during interesting moments (compared to rest and to non-interesting moments) reflects creative processes, which play an important role in music therapy.

However, there appears to be a difference in topographical distribution between the two moments of interest. Benedek et al. (2014) concluded that focused internal attention shows alpha power increases in the right parietal cortex. In the second MOI we observe a topography in which the right parietal alpha power seems to be decreased and the Traveler is focusing on sending messages to the unborn baby. Additionally, the Guide describes a strong feeling of mutuality with the Traveler during these passages (see section “General Comments on the GIM Session”). So, the inverse to what Benedek described may have been observed here: while focusing attention on internal mental imagery, the intentional direction of the imagery is turned outward and along with music and the feeling of mutuality while being in a therapeutic relationship alpha power in the right temporo-parietal cortex decreases. Further research is needed to investigate these ideas, including the role of creative ideation during therapy, in more detail.

#### Emotion: Frontal and Parietal Differences

*Proposition I: We expect differences between emotional processing during important moments and during the resting-state, and thus a difference in the alpha asymmetry (AA) measures*.

*More specifically, compared to the resting-state, we expect during MOIs more left-shifted frontal AA, possibly indicating positive emotional valence, and more right-shifted parietal AA, possibly indicating emotional intensity/arousal*.

*Proposition II: We expect FMT power to increase during positive emotional processing*.

Because previous studies have shown changes in frontal brain activity during and after receiving music therapy (Lem, 1998; Lee et al., 2012; Fachner et al., 2013; O'Kelly et al., 2013), we investigated a few propositions regarding frontal and also parietal brain activity during MOIs, which was compared to rest and MONI. Previous studies reported a frontal asymmetry shift toward positive emotional processing after listening to preferred music (Field et al., 1998) or during musical emotions (Schmidt and Trainor, 2001; Altenmuller et al., 2002). Whereas the Field et al. study was embedded in a therapeutic framework of treating depressed mothers and FAA was extracted from pre/post-listening resting-state recordings, our subjects were doing a GIM session and imaging while listening to music, which again is different to listening only conditions, as used in the studies by Altenmuller et al. (2002) and Schmidt and Trainor (2001).

Although the Traveler's asymmetry scores were more left-shifted (FAA) during MOIs compared to rest (which confirmed the FAA part of proposition I; see section “Emotion-Related Activity”), MONI FAA returned more positive frontal asymmetries (indicating a more positive processing) then during the MOIs. There were no differences between the MOIs for the Traveler. Schmidt and Trainor (2001) used increased relative right PAA as a marker of arousal and intensity of emotional processing, but found no clear evidence for representing intensity. In our study, the Guide's MOI PAA was less positive compared to rest (not confirming the PAA of proposition I). Further the Guide's FAA differed between the two MOIs and rest and P/FAA indicated more negative processing during the second MOI.

Interestingly emotion processing during MOIs was not that positive as proposed. This was also confirmed with FMT, which we expected to indicate increased positive emotional processing (Proposition II; see Aftanas and Golocheikine, 2001; Sammler et al., 2007; Nemati et al., 2019). In general, rest and MONI had higher FMT power indicating relaxation, while less power was observed during the MOIs and during Rest2.

Thus, MOI AA and FMT results returned a more “negative” overall emotional processing. This may reflect the therapeutic work on a potential harmful expected life event of a close relative. Even when experiencing a message from grandma and being connected to the family tree (a more positive image), still the possibility existed that the grandchild will not survive nor will the daughter-in-law. Further, the emotional processing of the Guide may reflect the empathic therapeutic concern for the Traveler and realizing and partly feeling the impact on the Traveler. A recent study indicated that right-frontal processing is related to empathic concern (Tullett et al., 2012), and we may speculate that the more negative asymmetry values of the therapist are related to this.

In contrast to previous studies investigating the processing of preferred, liked or disliked music in neutral laboratory settings (Eerola and Vuoskoski, 2013), in the real-world therapy there were real threads to be processed and coping with fear and anxiety was the reason to do the journey. Indeed, this processing is likely reflected in the F/PAA and FMT MOI measures of Guide and Traveler and the experts did identify the parts of the session where meaningful spontaneous imagery occurred that offered hope and promising change.

### Role of the Altered State of Consciousness (ASC)

In a former study, we reported a neurometric difference between the resting-state and the altered state induction as indicated by z-scores (compared to a normative EEG database) on the alpha range (Fachner et al., 2015). In this study, we found that during the induction of the ASC, the Traveler showed a more negative FAA compared to the resting-state. This suggests that the induction was effective and that the Traveler entered an altered state of consciousness, which in turn has to be kept in mind when considering imagery and emotion-related processing of the Traveler during the session.

GIM has evolved out of a pharmaco-supported psychotherapy setting in which the ASC has been evoked with strong psychedelic drugs (Bonny and Pahnke, 1972). However, Helen Bonny observed that inducing an ASC with deep relaxation methods and a subsequent journey in a reclined or supine position elicited sufficient imagery for the therapy session (Bonny, 1980a). Nevertheless, we know from recent research into MDMA or LSD that the vividness of imagery is stronger due to the breakdown of ego defense mechanisms (Carhart-Harris and Friston, 2010; Kaelen et al., 2015, 2016, 2018a,b) and the emergence of important imagery along with the music, for example in Amazonian Ayahuasca (De Rios, 1972; Shannon, 2011) yield strong imagery patterns with a culture specific taxonomy and noetic structure that may well yield a vast amount of important emerging imagery that can be used for therapy (Bonny, 1980a). Recent research investigating psychedelics have shown imagery enhancement under the influence (Kaelen et al., 2015, 2016, 2018a,b). However, the integration and conscious experience yielded with the relaxation induction methods can be controlled voluntarily while the imagery intensity under the influence may put the Traveler in a “floodlight state” that needs longer time to be processed and integrated (Grocke, 1999; Kaelen et al., 2018a,b). However, research into deep relaxation with music and imagery (Pfeifer et al., 2016) and reviewing common features of music and altered states stresses that setting, performance rites, suggestibility traits, and personal willingness to go into an altered state are of importance (Fachner, 2011). Thus, a proper investigation into the role of the ASC according to the induction method during these interventions is needed.

### Explorative vs. Naturalistic Design to Study Spontaneous Imagery

A recent review stressed “the importance of the ecological characteristic of the experiments as […] this is a key feature when investigating human interactions” (Acquadro et al., 2016, p. 9; see also Debener et al., 2012). In this objectivist case study, we addressed this demand for investigating ecological situations and explored the underlying neural correlates of a therapy process and tested a few propositions. Thus, the main intention was not to set up an experimental study on imagery and emotion in the brain, but to accompany a real-world music therapy session with EEG and video (which however lacks the experimental control typical for lab-based studies) (Fachner, 2014; 2016). This therapy took place to help a person cope with potential suffering, namely the life-threatening illness of a family member awaiting a baby, while we were interested in emerging meaningful imagery (see above) that is of pivotal importance for the patient in order to achieve change.

However, technological and methodological advances in hyperscanning and multi-modal data recording possibilities (Müller et al., 2013; Maidhof et al., 2014; Zamm et al., 2017) allowed for this exploratory dual-EEG study into shared imagery and emotion in the therapy process. Thus, a reflexive research process started, linking the first (subjective experience, imagery and emotions) and third (objective: physiological measures) person perspective (Fachner, 2014; Hunt, 2015; 2016; 2017).

Michel et al. (1993) and Lehmann et al. (1998) were studying spontaneous mentation and imagery by probing imagery and thought processes. Other more recent studies into the time-course of alpha power changes in creative ideation investigated continuous data in order to capture emerging thought elicited under experimental control (Schwab et al., 2014). Such experimental set-ups clearly yield in sum more defined spontaneous imagery or thought processes that can be repeated and therefore allow firmer inferences than can be drawn from a single trial during a real-world session.

Clearly, more repetitions of such case studies are needed that investigate focused questions about action mechanisms of arts-based interventions. However, in order to accompany an arts-based healing process, we have to adapt the tools that allow investigating brain processes *in situ* (Fachner, 2014; Hunt, 2015). Creative art process strives for unique results accounting all contingencies and situatedness and are not meant to be repeated, while scientific processes are unique in how they can and ought to be repeated. As we are researching healing processes, we have to accept the uniqueness of the therapeutic interaction (Fachner, 2017) while being able to observe these processes (for example, with EEG and video). After observing the processes in different ecological situations, we may then meta-analyze series of objectivist case studies in order to describe commonalities in procedures regarding emotion and imagery, turn-taking and co-activity, or other assorted phenomena of social interaction that are part of the therapeutic relationship. By doing so, we may also be able to describe a neural signature of what makes the therapeutic relationship in music therapy unique and at the same time comparable to the “sociophysiology” of other psychotherapeutic relationships (Schiepek, 2011, p. 351ff).

### Limitations

This is an explorative single case study investigating a real-life music therapy session. Therefore, findings need to be interpreted with caution and cannot be generalized. In a strict sense, this study cannot be repeated and findings not be replicated. Nevertheless, our initial observations can be addressed in future studies more specifically. For this, and to derive at an understanding of the mechanisms underlying music therapy, we believe a dual strategy consisting of series of case studies (allowing meta-analyses to find commonalities) in parallel to controlled experiments with healthy participants (allowing testing of hypotheses derived from case studies) can be a fruitful approach.

Both participants were listening to the same music, and thus any similarity in emotional processing could be in principle due to the common factor of music-evoked emotions in both participants. However, we also found several differences in emotional processing as well as imagery-processes between the participants. For example, whereas the FAA of the Traveler was more positive during therapeutically important moments, it did not differ for the Guide. Given the different roles of the participants and the dynamic relationship between musical intensity, the Traveler's imagery and the sharing with the Guide, we believe that a simple explanation in terms of commonly evoked musical emotions in both participants seems rather unlikely. In contrast, this study may call for a special awareness toward emotional peaks of intensity and close connection between musical intensity (profile) and (peak, pivotal) experiences. For future work, it is even conceivable to combine the moment-to-moment interaction with neurofeedback approaches (e.g., bio-guided music therapy; see Miller, 2011) to apply patient-specific peak thresholds as targets for emotion regulation and awareness.

One could argue that wearing EEG caps, being connected to an EEG amplifier and filmed could have influenced the authenticity and thus quality of the session (possibly influencing imagery, emotional processes, etc.). However, the Traveler remarked interestingly in the post-session talk (i.e., without being specifically asked about it by the researchers, which might have introduced a bias) to the Guide that she completely forgot about the whole “lab-like” situation, including wearing an EEG cap and the video camera—it appeared she was only realizing this situation after coming out of the ASC. This confirms ideas about the feasibility of music therapy *in-situ* EEG-hyperscanning studies that do not affect the therapy process itself significantly (Fachner, 2016).

Another limitation might be that both independent raters as well as Guide and Traveler were experienced GIM therapists. Therefore, the therapy, the selection of interesting moments and their descriptions were based on expert knowledge, given that Guide and Traveler are (international) colleagues and have more than 30 years of experience in music therapy. Spiro and Himberg (2016) pointed out that studies based only on the perspective of therapists may miss out on “other aspects what may be going on” (p. 4) and therefore expert bias is potentially accumulated here (see section “Moment of Interest (MOI) Identification”). On the other hand, the addition of two external raters not involved in the therapy and the reports from the Traveler—being experienced in interpreting and reflecting about therapy processes herself—seems to be, at least in this instance, an appropriate strategy to gain insights into what happened during the session and to achieve a higher content validity of the MOIs. However, this expert knowledge might have influenced the flow and emergence of the imagery and emotional processing, i.e., access to important imagery might be enhanced in experienced Travelers; nevertheless, it is likely that a personal pivotal meaning for the experienced Traveler was the same as it would have been for any other Traveler or client (Grocke, 1999). Still, this calls for further research into emerging imagery and the role of background knowledge and different relationship situations (Bonde and Beck, 2019; Dukic et al., 2019).

We used well-known indices of cortical emotional processing that have also been used in a clinical context in numerous studies (for overviews, see e.g., Allen et al., 2004; Mitchell et al., 2008; Allen and Reznik, 2015; Smith et al., 2017) and also in music and emotion studies (Schmidt and Trainor, 2001; Altenmuller et al., 2002; Sammler et al., 2007). However, future studies could employ more recently developed indices of brain-to-brain coupling. In the music domain, these measures have been mainly used to investigate interpersonal action coordination (Sänger et al., 2013; D'Ausilio et al., 2015; Zamm et al., 2017). Given the importance of emotional processes in social interactions including music performance (Acquadro et al., 2016), it will be interesting to develop other measures of brain-to-brain coupling related to emotion, which have the added benefit of a higher temporal resolution due to the time-frequency decomposition of the data.

Another limitation is that differences in FAA between resting-state (no interaction) and moments during the session might be only due to differences in the social interaction including verbalizations, and do not reflect differences in emotional processing. However, when comparing the FAA during MOIs and MONI, where, importantly, a comparable interaction (including verbal interactions) took place, the FAA differed, suggesting that not only physical differences have contributed to the change in alpha asymmetries between resting-state and MOIs. To clarify this issue future studies could compare similar social interactions but differing in their emotional (verbal) content.

Whether age has an influence on our FAA results cannot be estimated given the sparse research available with FAA and music and older people. One study focusing on neurofeedback training with depressed older people (Ramirez et al., 2015) reported a decrease of relative left alpha activity indicating an improvement of their depression condition. Future studies may investigate potential effect of age on markers of emotional processing.

## Conclusions

In this explorative objectivist case study of Guided Imagery and Music, a Traveler was working with the assistance of a Guide on a threatening problem of losing family members. In this case, we observed that intense emotional processing of imagery in GIM has a temporal dynamic and was shared with the Guide. The EEG-derived biomarkers, indexing imagery- and emotion-related processes, suggested that during the important moments of therapy, emotions were not positive, pleasurable and relaxed (even though the Traveler was in an ASC) as opposed to our proposition. The significant differences between resting-state and therapy in the emotional valence and intensity, as measured with frontal and parietal alpha asymmetries as well as frontal midline theta power, confirm that both were dealing with negative emotions and anxiety that had to be contained in the interpersonal process. Nevertheless, the therapeutic setting offered a secure base to work on negative emotions, fear, and anxiety, and allowed the Traveler to develop hope and a perspective that change is still possible and that there may be a good end.

During a pivotal moment captured in this study, in which an imagined significant person delivered a message of hope (“not to worry too much”), we could study in detail how emotional processing changed from negative to positive valence and how Traveler and Guide shared the dynamics of this emotional processing, in particular how the Guide's emotional peak processing followed the one of the Traveler until the message was verbalized by the Traveler.

Even though this is an explorative case study and findings need to be interpreted with caution, we believe that the methodological approach of combining dual-EEG and detailed video-based and qualitative data is a promising approach for future research in this domain.

By analyzing the moment-to-moment interaction in music therapy, we were able to observe how frontal and parietal asymmetry peaks aligned with the situated cognition of Traveler and Guide along the emotional contour of the music, representing the highs and lows during the therapy process. This might have implications for a further understanding of the underlying mechanisms of music therapy and may contribute in the future to reveal what interventions (and when delivered) may be more effective.

## Ethics Statement

This study was carried out in accordance with the recommendations of Code of practice for applying for ethical approval at Anglia Ruskin University with written informed consent from all subjects. All subjects gave written informed consent in accordance with the Declaration of Helsinki. The protocol was approved by the ARU Departments Research Ethics Panel.

## Author Contributions

JF and CM drafted the manuscript, developed methods, design of the study, and conducted measurements and analysis. DG and IN contributed to design, procedure and draft. GTr and LB contributed to design and draft. GTu contributed to the methods and LB initiated the EEGIM research.

### Conflict of Interest Statement

DG, IN, GTr, and LB are GIM therapists. The authors declare that the research was conducted in the absence of any commercial or financial relationships that could be construed as a potential conflict of interest.

## Footnotes

^1^Translation from French as to http://www3.cpdl.org/wiki/index.php/L%27adieu_des_bergers_(Hector_Berlioz)

^2^Note that the part of the EEG segment containing the swallowing artifact has been rejected (duration: ca. 1.7 s), which resulted in 3 missing data points in the FAA time series. These have been linearly interpolated. Because the peak, measured from zero-crossing before and after the peak, consisted of 11 data points, it appears likely that the observed peak, even though partly based on interpolated and filtered data, reflects a peak in FAA and not just an artifact due to the interpolation.

## Supplementary Material

Video 1 Video excerpt of the identified Moment of Interest 1 - Segment 1 of the GIM session. The Guide is sitting on the left side, while the Traveler is laying down with eyes closed (right side). In this excerpt, the Traveler describes her imagery in which her grandmother told her “not to worry so much”. The music during this segment is H. Berlioz: L'enface du Christ (Shepard's Farewell). See Figure 1 for the time course of the frontal alpha asymmetry and Table 3.

## Abbreviations

AA: Alpha Asymmetries
ASC: Altered States of Consciousness
EEG: Electroencephalogram
FAA: Frontal Alpha Asymmetry
FMT: Frontal Midline Theta
GIM: Guided Imagery and Music
MOI: Moments of Interest
MONI: Moments of No-Interest
MTG: Middle Temporal Gyrus
PAA: Parietal Alpha Asymmetry
ROI: Region of Interest.
